# Infant formula with added *Lacticaseibacillus rhamnosus* GG supported adequate growth and was well tolerated in healthy term infants: a randomized controlled trial

**DOI:** 10.3389/fped.2024.1456607

**Published:** 2024-10-23

**Authors:** Carol Lynn Berseth, Michael Yeiser, Cheryl L. Harris, Jennifer N. Kinnaman, Victoria Lappin, Jennifer L. Wampler, Weihong Zhuang, Jon Vanderhoof

**Affiliations:** ^1^ Medical Sciences, Reckitt | Mead Johnson Nutrition, Evansville, IN, United States; ^2^ Owensboro Pediatrics, Owensboro, KY, United States; ^3^ Nutrition Sciences, Reckitt | Mead Johnson Nutrition, Evansville, IN, United States; ^4^ Clinical Research, Research & Development, Reckitt, Hull, United Kingdom; ^5^ Gastroenterology, Boston Children’s Hospital, Boston, MA, United States

**Keywords:** term infant, pediatric nutrition, infant formula, *Lacticaseibacillus rhamnosus* GG, healthy growth

## Abstract

**Introduction:**

Lacticaseibacillus rhamnosus GG (LGG) is a well-studied probiotic with a history of safe use.

**Methods:**

In this double-blind, prospective study, growth and tolerance were evaluated in healthy term infants randomized to: marketed, routine intact cow's milk protein-based formula (Control, *n* = 172) or a similar investigational formula with added LGG (INV-LGG, *n* = 179; 10^6^ CFU LGG®/g powder) from 14 to 120 days of age. Anthropometrics, stool characteristics, fussiness, and gassiness were evaluated through Day 120. Medically confirmed adverse events were recorded throughout the study period. The primary outcome was rate of weight gain from Day 14–120.

**Results:**

Of 351 infants enrolled, 275 completed (Control, *n* = 131; INV-LGG, *n* = 144). No significant group differences in rate of weight gain from Day 14–120 were detected. Study formula acceptance and tolerance was good with no significant differences in study discontinuation due to study formula or parent-reported gassiness, stool frequency, or stool consistency; however mean fussiness relative to normal was significantly lower for INV-LGG vs Control at Days 60 and 90.

**Discussion:**

In healthy term infants, a routine intact cow's milk protein-based formula with added LGG supported adequate growth and was well tolerated. Further studies are needed to evaluate potential benefits for fussiness and efficacy outcomes.

**Clinical Trial Registration:**

Clinicaltrials.gov, identifier (NCT01897922).

## 1 Introduction

The gut microbiome plays an important role in the health and development of an individual. Within the intestine, the microbiota influences nutrient metabolism (such as through short-chain fatty acids and some vitamin production), improves barrier function, reduces pathogen colonization, and stimulates the immune system. It is recognized that differences in dietary patterns early in life, such as breastfeeding or formula feeding, modulate the microbiota. For example, infants receiving human milk typically have a more stable microbiota population characterized by a higher relative abundance of Bifidobacteria, *Lactobacillus*, and *Staphylococcus*, in contrast to formula-fed infants, who demonstrate a higher alpha diversity (1–3). Differences are thought to be driven, in part, by the consumption of the microbiota present in human milk as well as human milk oligosaccharides (HMOs), the indigestible prebiotics naturally found in human milk that selectively feed the beneficial bacteria in the gut (4–9).

As infant formulas are inspired by the composition and benefits of human milk, the addition of specific prebiotics and probiotics to infant formula may be one method of helping to support the microbiota of formula-fed infants. We have previously evaluated infant formula with an added prebiotic blend of polydextrose (PDX) and galacto-oligosaccharides (GOS) (1:1 ratio, 4 g/L) in healthy term infants. The formula supported adequate growth, promoted bifidobacteria and lactobacilli colonization closer to that of breastfed infants, and softened stools as compared with infants who received a formula without PDX and GOS (10–13). The same prebiotic blend added to a follow-on formula induced a pattern of more frequent and softer stools in toddlers, further indicating a beneficial effect of the prebiotic blend on gut health (14).

Infant formula with added *Lacticaseibacillus rhamnosus* GG (LGG®; formerly *Lactobacillus* GG and commonly known as “LGG”) has also been observed to be well-tolerated and support adequate growth in infants (15, 16). LGG transiently colonizes the intestine in healthy infants, with suggested benefits including the augmentation of the local immune defense and contribution to an increased response to vaccinations through stimulation of antibody production (17–20). In infants with cow's milk allergy, the use of extensively hydrolyzed casein-based formula with added LGG reduces inflammation in the skin and gastrointestinal (GI) tract and induces oral tolerance at a younger age (21–24). Dietary LGG has also been shown to influence the infant microbiome by increasing the abundance of certain Bifidobacteria and butyrate-producing organisms (25–27).

Whereas the prebiotic blend of PDX and GOS and the probiotic LGG have been evaluated separately, with demonstrated benefits for infants as described previously, the combination of them has not been assessed. The present study was designed to evaluate growth and tolerance in healthy term infants receiving an investigational intact cow's milk protein-based formula that had LGG and an added prebiotic blend of PDX and GOS through 120 days of age compared to a marketed intact cow's milk protein-based formula that had the prebiotic blend only.

## 2 Materials and methods

### 2.1 Study design and population

In this multicenter double-blind randomized controlled parallel-designed prospective study, mothers who had decided to exclusively provide infant formula were screened for study eligibility. Parents/guardians provided written informed consent prior to enrollment. Participants were healthy term 12- to 16-day-old infants. Eligibility also included: singleton births at 37–42 weeks' gestational age, birth weight of ≥2500 g, and solely formula-fed for at least 24 h prior to randomization. Exclusion criteria included the following: a history of underlying metabolic or chronic disease, including immunosuppression and congenital malformation; feeding difficulties or formula intolerance at randomization; <98% of birth weight at randomization; and being large for the gestational age from a mother who was diabetic at childbirth. Participants were enrolled between July and December 2013 at 23 study sites in the USA until enrollment was met (Clinicaltrials.gov registration: NCT01897922).

The study sponsor created computer-generated randomization schedules stratified by sex and provided in sealed consecutively numbered envelopes to each study site. Study formula was assigned by opening the next sequential envelope from the appropriate set at the study site. Study formulas were each designated by two unique codes known only to the sponsor. Neither the product labels nor the sealed envelopes allowed direct unblinding by the study site. The personnel responsible for monitoring the study were also blinded to the study product identification. The study code for an individual participant could be broken in the event of a medical emergency in which knowledge of the study formula was critical to the participant's management. In this study, it was not necessary to break the study code prematurely.

Participants were randomly assigned to receive one of two study formulas (Mead Johnson Nutrition, Evansville, IN, USA), Control (marketed intact cow's milk protein-based infant formula) or a similar formula with added *L. rhamnosus* GG (INV-LGG) (10^6^ CFU LGG®/g powder; Chr. Hansen Holding A/S, Denmark), from 14 to 120 days of age (Table 1). Both study formulas had docosahexaenoic acid (DHA at 17 mg/100 kcal), arachidonic acid (ARA at 34 mg/100 kcal), and a prebiotic blend of PDX and GOS (4 g/L; 1:1 ratio).

**Table 1:** Nutrient composition per 100 kcal.

Nutrient	Study formulas (target values)	
	Control	INV-LGG^a^
Protein (g)	2.1	2.1
Fat (g)	5.3	5.3
Total carbohydrate^b^ (g)	11.2	11.2
Arachidonic acid (mg)	34	34
Docosahexaenoic acid (mg)	17	17
Vitamin A (IU)	300	300
Vitamin D (IU)	60	60
Vitamin E (IU)	2	2
Vitamin K (μg)	9	9
Thiamin (μg)	80	80
Riboflavin (μg)	140	140
Vitamin B6 (μg)	60	60
Vitamin B12 (μg)	0.3	0.3
Niacin (μg)	1,000	1,000
Folic acid (μg)	16	16
Pantothenic acid (μg)	500	500
Biotin (μg)	3	3
Vitamin C (mg)	12	12
Choline (mg)	24	24
Inositol (mg)	6	6
Calcium (mg)	78	78
Phosphorus (mg)	43	43
Magnesium (mg)	8	8
Iron (mg)	1.8	1.8
Zinc (mg)	1	1
Manganese (μg)	15	15
Copper (μg)	75	75
Iodine (μg)	15	15
Selenium (μg)	2.8	2.8
Sodium (mg)	27	27
Potassium (mg)	108	108
Chloride (mg)	63	63

^a^ Added LGG at 10^6^ CFU/g powder.

^b^ Available carbohydrate for Control or INV-LGG, 10.6 g; prebiotic oligosaccharides = 0.6 g {source, prebiotic blend of polydextrose (PDX, Litesse® Two Polydextrose; Danisco) and galacto-oligosaccharides [GOS, (Vivinal® GOS Galacto-oligosaccharide; Friesland Foods Domo)]} (1:1 ratio, 4 g/L).

### 2.2 Study objectives and outcomes

The objective was to evaluate growth and tolerance in healthy term infants. Birth anthropometric measures (body weight, length, and head circumference) were obtained from participant birth records. At all study sites, anthropometrics were recorded at days 14, 30, 60, 90, and 120 using the following standardized procedures. At each study visit, body weight was measured using a study-designated calibrated pediatric balance (nearest g or oz), body length was measured (to the nearest half centimeter or quarter of an inch) using a recumbent pediatric stadiometer, and head circumference was measured (to the nearest half centimeter or quarter of an inch) using a flexible non-stretchable tape provided by the study sponsor. Parents completed a baseline recall of tolerance (fussiness and gassiness) and stool characteristics (frequency and consistency) at study enrollment and a 24 h recall of study formula intake, tolerance (fussiness and gassiness), and stool characteristics (frequency and consistency) at subsequent study visits. Responses were scaled for the amount of gas (0–4, corresponding to none, slight, moderate, or excessive), fussiness (0–4, corresponding to not fussy, slightly, moderately, very, or extremely fussy), and stool consistency (1–5, corresponding to hard, formed, soft, unformed, or seedy, watery). Adverse events (AEs) were coded according to specific events and categories of the body system.

### 2.3 Ethics

The study was conducted according to the guidelines of the Declaration of Helsinki (including the October 1996 amendment). The research protocol (protocol #3385-2) and informed consent forms were approved by the Schulman Associates Institutional Review Board (SAIRB, Cincinnati, OH, USA; date of approval: 22 March 2013, IRB #201301563). The study complied with good clinical practices.

### 2.4 Statistical methods

The primary outcome was the rate of weight gain from 14 to 120 days of age. The sample size was chosen to detect a clinically relevant weight gain difference of 3 g/day (80% power; *α* = 0.05; one-tailed) from 14 to 120 days of age. Assuming a standard deviation of 6.5 g/day for male participants and 5.5 g/day for female participants, 59 male and 43 female participants per study group were required to complete the study. Analysis of variance (ANOVA) was used to assess the rate of weight gain at 30, 60, 90, and 120 days of age calculated for each participate by fitting a linear regression of weight on age. For females, owing to variation detected at enrollment, a covariate of “head circumference at enrollment” was used to analyze the achieved head circumference and growth rate. Mean rate of weight gain by sex and study formula group was were compared using one-tailed tests, as outlined in the American Academy of Pediatrics (AAP) Task Force on Clinical Testing of Infant Formulas (28). All other comparisons were two-tailed. Secondary outcomes included: formula intake and stool frequency (analyzed by ANOVA); stool consistency, fussiness, and gas [analyzed using the Cochran–Mantel–Haenszel (CMH) row means score test]; and the incidence of adverse events (analyzed by Fisher's exact test). All tests were conducted at *α* = 0.05. Statistical analyses were performed using SAS® software (version 9.2; SAS Institute, Cary, NC, USA).

## 3 Results

### 3.1 Participants

**Figure 1:**
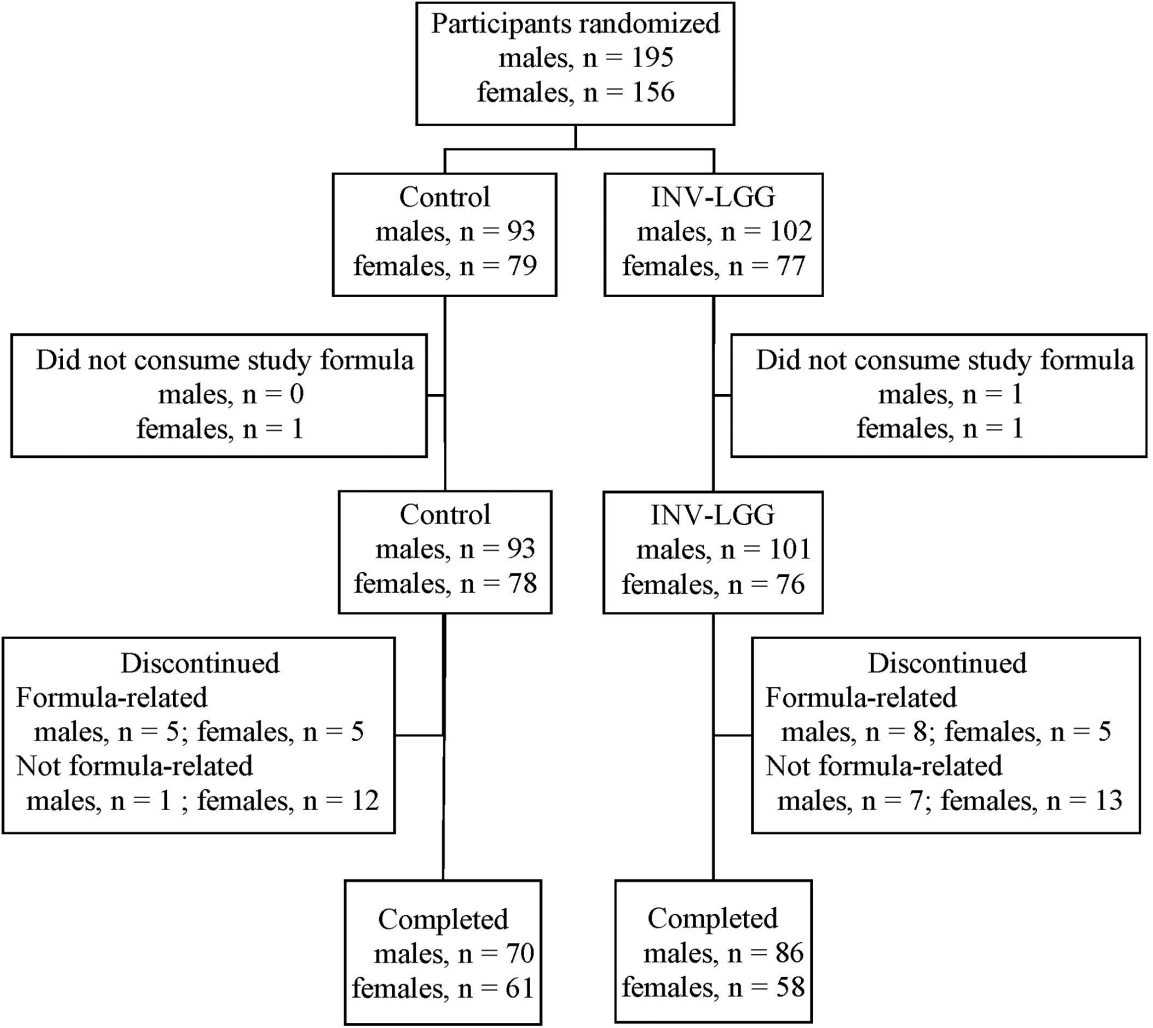
Allocation of study participants.

A total of 351 infants (Control, *n* = 172; INV-LGG, *n* = 179) were enrolled and randomized (Figure 1). Participants who were randomized but consumed no study formula were not included in subsequent analyses (Control, *n* = 1; INV-LGG, *n* = 2). With the exception of head circumference in females, no group differences in body weight, length, or head circumference by sex were observed at study enrollment (Table 2). Birth anthropometric measures as well as sex, race, ethnic distribution, and a family history of allergy were similar among groups. No statistically significant group differences were detected for overall study discontinuation (Control, *n* = 40, 23%; INV-LGG, *n* = 33, 19%) or discontinuation related to study formula (Control, *n* = 10, 6%; INV-LGG, *n* = 13, 7%). In the total study population, 20 participants (6%) discontinued due to formula intolerance as determined by the study investigator, with fussiness (Control, *n* = 5; INV-LGG, *n* = 5) and gas (Control, *n* = 3; INV-LGG, *n* = 4) as the most common indicators. A total of 275 participants completed the study (Control, *n* = 131; INV-LGG, *n* = 144).

**Table 2:** Infant characteristics at enrollment.

	Control	INV-LGG	*P*
Total number of participants	171	177	
Sex, *n* (%)			0.666
Female	78 (46)	76 (43)	
Male	93 (54)	101 (57)	
Race, *n* (%)			0.424
Black	20 (12)	21 (12)	
White	134 (79)	146 (82)	
Other	16 (9)	10 (6)	
Ethnicity, *n* (%)			0.845
Hispanic	13 (8)	15 (8)	
Not Hispanic	158 (92)	162 (92)	
Family history of allergy	73 (43)	81 (46)	0.590
Males^a^			
Weight (g)	3,679.0 ± 44.8	3,700.0 ± 43.0	0.736
Length (cm)	52.1 ± 0.2	52.4 ± 0.2	0.379
Head circumference (cm)	36.3 ± 0.1	36.3 ± 0.1	0.940
Females^a^			
Weight (g)	3,577.3 ± 43.9	3,473.2 ± 44.5	0.098
Length (cm)	51.7 ± 0.2	51.3 ± 0.2	0.221
Head circumference (cm)	36.0 ± 0.1	35.4 ± 0.1^a^	0.007

^a^ Mean ± standard error (SE).

### 3.2 Growth

The required number of male and female participants completed study feeding through 120 days of age. Post-study power calculations indicated that this study was powered to detect a 3 g/day difference in weight gain from 14 to 120 days of age for male (94%) and female (93%) participants at the levels indicated. No statistically significant group differences in the primary outcome, the rate of weight gain from day 14 to day 120, were detected by sex (Table 3). No statistically significant group differences were detected for weight, length, or head circumference growth rates by sex for any measured range for growth rates analyzed from day 14 to day 120.

**Table 3:** Weight, length, and head circumference growth rates from 14 days to 30, 60, 90, and 120 days of age.

Day	Group (*n*)	Growth rate^a^		
		Weight (g/day)	Length (cm/day)	Head circumference^b^ (cm/day)
Males				
30	Control (88)	41.2 ± 1.3	0.16 ± 0.009	0.10 ± 0.004
	INV-LGG (97)	43.0 ± 1.2	0.14 ± 0.009	0.10 ± 0.004
60	Control (74)	37.2 ± 0.9	0.13 ± 0.004	0.07 ± 0.002
	INV-LGG (88)	38.0 ± 0.8	0.13 ± 0.004	0.07 ± 0.002
90	Control (73)	33.9 ± 0.8	0.12 ± 0.003	0.06 ± 0.002
	INV-LGG (87)	34.1 ± 0.7	0.12 ± 0.002	0.06 ± 0.001
120	Control (70)	31.3 ± 0.7	0.11 ± 0.002	0.06 ± 0.001
	INV-LGG (86)	31.6 ± 0.6	0.11 ± 0.002	0.06 ± 0.001
Females				
30	Control (76)	36.2 ± 1.3	0.13 ± 0.008	0.08 ± 0.005
	INV-LGG (72)	34.5 ± 1.3	0.12 ± 0.009	0.09 ± 0.005
60	Control (68)	31.0 ± 1.0	0.12 ± 0.004	0.07 ± 0.002
	INV-LGG (64)	29.5 ± 1.0	0.11 ± 0.004	0.07 ± 0.002
90	Control (64)	28.7 ± 0.8	0.11 ± 0.002	0.06 ± 0.001
	INV-LGG (60)	27.1 ± 0.8	0.11 ± 0.002	0.06 ± 0.001
120	Control (61)	27.3 ± 0.7	0.10 ± 0.002	0.05 ± 0.001
	INV-LGG (58)	25.7 ± 0.7	0.10 ± 0.002	0.05 ± 0.001

^a^ Mean ± standard error (SE).

^b^ Adjusted for head circumference at enrollment (females only).

**Figure 2:**
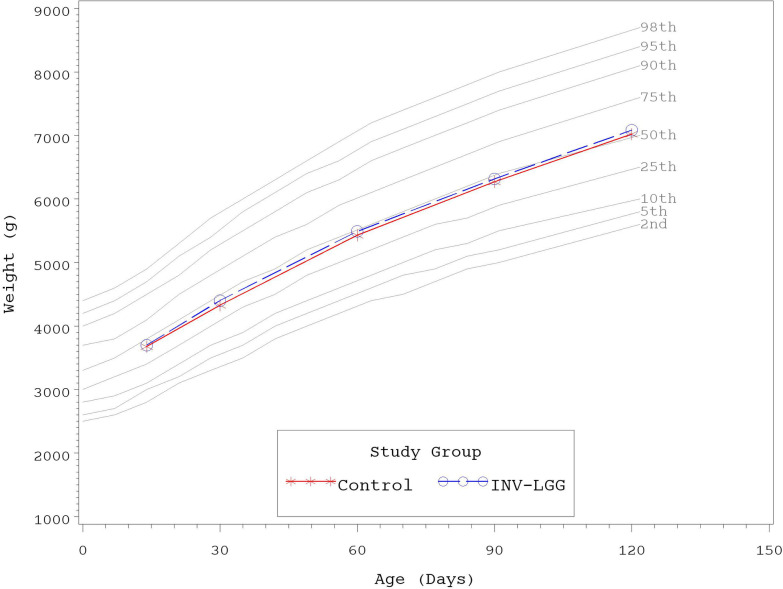
Mean achieved weights with WHO growth standard percentiles (2–98) from 14 to 120 days of age (males).

**Figure 3:**
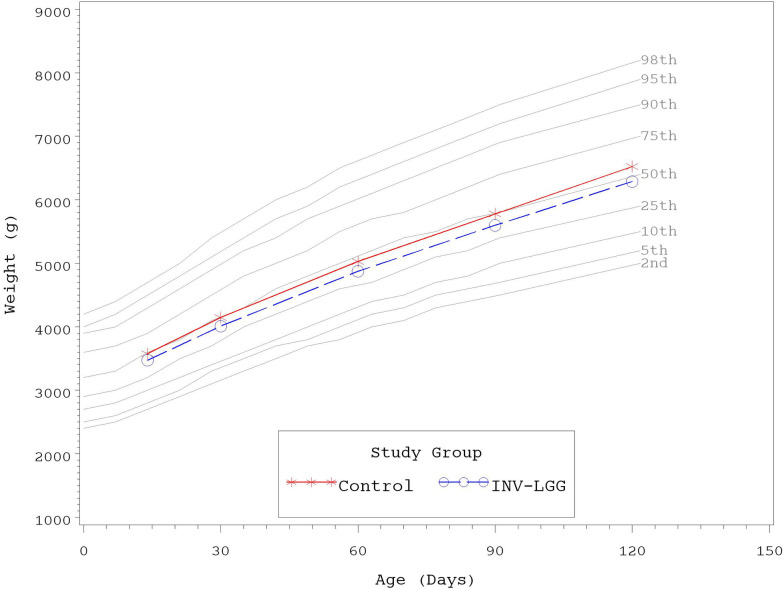
Mean achieved weights with WHO growth standard percentiles (2–98) from 14 to 120 days of age (females).

**Figure 4:**
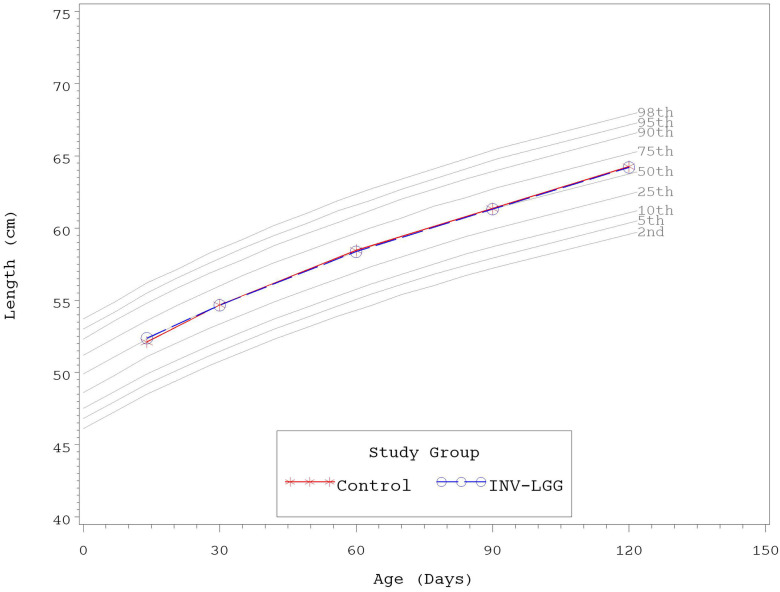
Mean achieved lengths with WHO growth standard percentiles (2–98) from 14 to 120 days of age (males).

**Figure 5:**
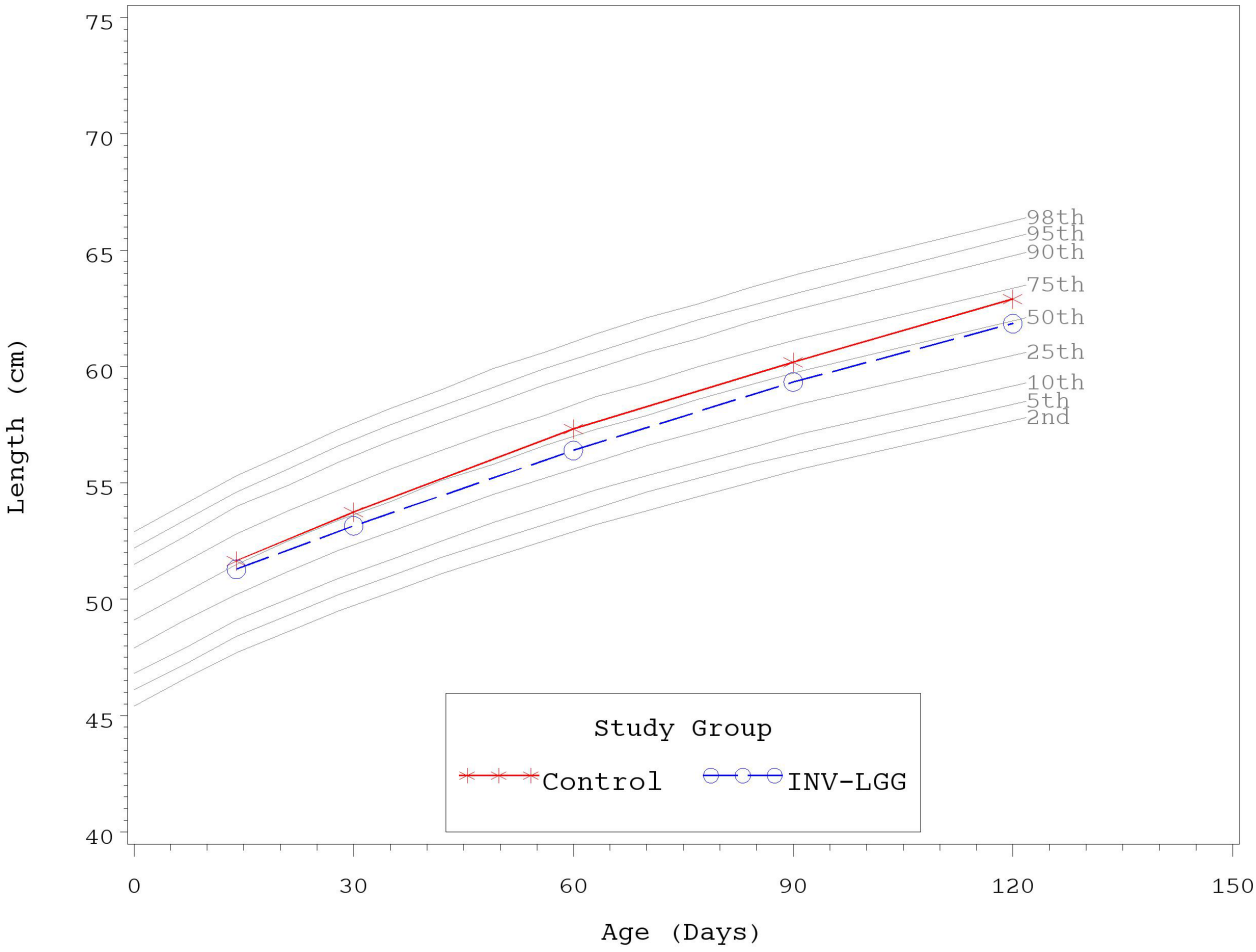
Mean achieved lengths with WHO growth standard percentiles (2–98) from 14 to 120 days of age (females).

No significant group differences were detected in the mean achieved weight or length for males at any measured time point (Table 4). For females, significant differences were observed, with a higher mean achieved weight at day 120 and a higher mean achieved length at days 60, 90, and 120 in the Control compared with the INV-LGG group. However, no significant group differences were detected in weight-for-length z-scores at any time point assessed, reflecting appropriate overall growth. In addition, the mean achieved weight for males (Figure 2) and females (Figure 3) plotted on the World Health Organization (WHO) weight-for-age standard growth chart (29, 30) fell between the 25th and 75th percentiles. Mean achieved lengths for males (Figure 4) plotted along the 50th percentile and for females (Figure 5) plotted within the 25th and 75th percentiles of the WHO length-for-age standard growth chart at all measured time points. No differences in achieved head circumference were detected for males. With the exception of differences detected for females at enrollment (day 14) that persisted at day 30, no group differences by sex in the mean achieved head circumference were detected at any other time point.

**Table 4:** Achieved weight, length, head circumference (HC), and weight-for-length z-scores at days 30, 60, 90, and 120 for males and females.

Day	Group (*n*)	Achieved growth^a^			Z-score Weight-for-length^a^
		Weight (g)	Length (cm)	HC (cm)	
Males					
30	Control (87)	4,327 ± 52	54.7 ± 0.2	37.9 ± 0.1	−0.41 ± 0.12
	INV-LGG (96)	4,401 ± 50	54.7 ± 0.2	37.9 ± 0.1	−0.19 ± 0.11
60	Control (73)	5,431 ± 68	58.5 ± 0.2	39.8 ± 0.1	−0.26 ± 0.12
	INV-LGG (87)	5,490 ± 62	58.4 ± 0.2	39.8 ± 0.1	−0.07 ± 0.11
90	Control (72)	6,273 ± 80	61.4 ± 0.2	41.3 ± 0.2	−0.16 ± 0.12
	INV-LGG (85)	6,318 ± 74	61.3 ± 0.2	41.1 ± 0.1	−0.06 ± 0.11
120	Control (67)	7,024 ± 91	64.3 ± 0.3	42.5 ± 0.1	−0.16 ± 0.13
	INV-LGG (86)	7,085 ± 80	64.2 ± 0.2	42.4 ± 0.1	−0.01 ± 0.11
Females					
30	Control (74)	4,147 ± 49	53.7 ± 0.2	37.2 ± 0.1^c^	−0.24 ± 0.11
	INV-LGG (69)	4,011 ± 51	53.2 ± 0.2	36.8 ± 0.1	−0.23 ± 0.12
60	Control (65)	5,028 ± 61	57.3 ± 0.2^c^	39.0 ± 0.1	−0.31 ± 0.15
	INV-LGG (63)	4,874 ± 61	56.4 ± 0.2	38.7 ± 0.1	−0.15 ± 0.15
90	Control (64)	5,781 ± 71	60.2 ± 0.2^b,c^	40.3 ± 0.1	−0.26 ± 0.14^b^
	INV-LGG (59)	5,599 ± 74	59.3 ± 0.3	40.0 ± 0.1	−0.23 ± 0.15
120	Control (57)	6,524 ± 81^c^	62.9 ± 0.3^c^	41.5 ± 0.1	−0.12 ± 0.14
	INV-LGG (53)	6,289 ± 84	61.9 ± 0.3	41.2 ± 0.1	−0.11 ± 0.14

^a^ Mean ± standard error (SE).

^b^ *n* = 63 for length and z-score measures.

^c^ Control vs. INV-LGG significantly different (*P* < 0.05).

### 3.3 Tolerance

At enrollment, parents reported that gassiness, fussiness, and stool characteristics were similar between the study groups. No statistically significant group differences were detected in parent-reported mean study formula intake (fluid oz/day) by sex at any time point assessed (Table 5). No significant differences in gassiness (including gassiness relative to normal), stool frequency, or stool consistency at days 30, 60, 90, and 120 were detected. Although no significant difference was detected between the study formula groups in parental-rated fussiness relative to normal at day 30 or day 120, significant differences were detected at day 60 (*p* = 0.017) and day 90 (*p* = 0.009). Compared with the Control group, at these time points, the INV-LGG group had a higher proportion of infants that were less fussy than normal and a lower proportion of infants that were more fussy than normal (Table 6).

**Table 5:** Study formula intake (fluid oz/day) at days 30, 60, 90, and 120 for males and females.

Day	Group (n)	Mean ± SE	*P*
Males			
30	Control (80)	27.4 ± 0.8	0.468
	INV-LGG (95)	28.2 ± 0.7	
60	Control (72)	31.8 ± 0.9	0.698
	INV-LGG (86)	32.3 ± 0.8	
90	Control (72)	34.6 ± 1.1	0.948
	INV-LGG (83)	34.7 ± 1.0	
120	Control (66)	36.9 ± 1.0	0.991
	INV-LGG (86)	36.9 ± 0.9	
Females			
30	Control (72)	28.5 ± 0.8	0.333
	INV-LGG (65)	27.4 ± 0.8	
60	Control (65)	32.1 ± 1.0	0.092
	INV-LGG (60)	29.7 ± 1.1	
90	Control (64)	35.4 ± 1.5	0.817
	INV-LGG (58)	34.9 ± 1.6	
120	Control (57)	37.0 ± 1.3	0.566
	INV-LGG (53)	35.9 ± 1.4	

**Table 6:** Fussiness relative to normal at days 30, 60, 90, and 120.

Day	Group	Fussiness relative to normal, *n* (%)			*P*
		Less fussy than normal	Same level of fussiness	More fussy than normal	
30	Control	15 (10)	113 (74)	24 (16)	0.228
	INV-LGG	8 (5)	124 (78)	28 (18)	
60	Control	7 (5)	104 (76)	26 (19)	0.017
	INV-LGG	14 (10)	117 (80)	15 (10)	
90	Control	8 (6)	100 (74)	28 (21)	0.009
	INV-LGG	16 (11)	110 (78)	15 (11)	
120	Control	14 (11)	90 (73)	20 (16)	0.134
	INV-LGG	18 (13)	109 (78)	12 (9)	

No group difference was detected (Control, *n* = 140, 82%; INV-LGG, *n* = 147, 83%) in the number of participants who reported at least one adverse event. The most commonly reported category for AEs included Gastrointestinal; Ears, Nose, and Throat; and Respiratory. Within the Gastrointestinal category, the most commonly reported AEs were gastroesophageal reflux, gas, and constipation. There were no significant differences in the frequency of AEs reported in the Control and INV-LGG groups. Any medically confirmed AE was considered serious if it resulted in death, was life-threatening, required inpatient hospitalization or the prolongation of existing hospitalization, resulted in persistent or significant disability/incapacity, or was a congenital anomaly/birth defect. Site physicians evaluated all serious adverse events to determine causality. During the study, 20 participants experienced serious adverse events (Control, 12, 7%; INV-LGG, 8, 5%), which were all considered unrelated to the study formula.

## 4 Discussion

This study demonstrated that a routine intact protein cow's milk-based formula with added probiotic LGG was safe and well-tolerated when fed to healthy term infants from 14 to 120 days of age. No significant group differences were observed for the weight, length, or head circumference growth rates by sex for any age range from 14 to 120 days of age. Although several statistically significant differences were detected in the mean achieved weight and length in females, no significant group differences were detected in weight-for-length z-scores at any time point assessed, demonstrating overall good growth with body weight in proportion to the attained length. For both study groups, the mean achieved weights and lengths plotted within the 25th and 75th percentiles and the mean achieved head circumferences plotted within the 50th and 75th percentiles of the WHO growth standards at all measured time points. The results are also aligned with previous reports demonstrating adequate growth and tolerance of dietary prebiotic and probiotic use in healthy term infants, including the addition of LGG to extensively or partially hydrolyzed protein formulas (15, 16) and the prebiotic blend of PDX and GOS in intact cow's milk protein-based formulas (10, 12, 13).

Overall study formula acceptance and tolerance was good. No differences in study discontinuation due to study formula were detected. No significant differences were observed in parent-reported study formula intake as well as gassiness, stool frequency, or stool consistency throughout the study period; however, compared with the Control, the INV-LGG group had a significantly higher proportion of infants that were less fussy than normal and a significantly lower proportion that were more fussy than normal at 60 and 90 days of age. Data regarding the overall impact of probiotics on crying and restlessness in infants have been mixed (31). The results in the current study in term infants are consistent with reports of significantly fewer excessive criers among preterm infants receiving LGG vs. placebo in a 60-day trial (32). Symptom management has also been demonstrated in other populations, such as infants with cow's milk allergy, in which the use of formulas with added LGG improved GI and skin health outcomes (21, 22, 33). Similarly, daily crying time improved in breastfed colicky infants after receiving dietary LGG over a 28-day period (34). In colicky infants receiving LGG vs. placebo over a 4-week period, a significant decrease in crying was demonstrated from parental interviews (although this effect was not replicated in the validated parental diaries) (35). However, other studies have found little impact on colic prevention or symptom management using dietary LGG (36, 37). Studies have previously suggested that differences may exist in the microbiome in colicky infants (38–40). As this study was designed to assess growth and safety in healthy infants, a larger more directed study would need to be conducted to better elucidate beneficial efficacy outcomes and potential mechanisms of action beyond the initial tolerance outcomes in the current study.

Strengths of the study include the double-blind randomized controlled design. In addition, the formula was fed as a sole source of nutrition, allowing for the examination of growth and tolerance due to formula intake during this time period. However, limitations of the study include that it was not designed to examine efficacy outcomes such as fussiness, in which case a detected statistical difference could readily be considered clinically meaningful. As the study was primarily carried out to assess growth and tolerance, it was conducted in otherwise healthy infants, and this effect may need to also be explored further in a population with fussiness or colic. Moreover, as the study examined the addition of LGG to a formula with added prebiotics, it may be that that this effect on fussiness could be mediated via the microbiota; yet, the fecal microbiota was not assessed in this study to understand whether any changes occurred that could have contributed to the effect.

## 5 Conclusions

The results of the current study demonstrate that routine intact cow's milk protein-based formula with the added PDX and GOS blend and LGG supported adequate growth and was well-tolerated in healthy term infants. This is consistent with previous data examining the PDX and GOS prebiotic blend and LGG independently. This study also highlighted a potential benefit for fussiness. Additional studies are needed to explore the effects on the microbiota and efficacy outcomes further.

## Data Availability

The datasets presented in this article are not readily available because de-identified participant data from the final research dataset used in the published article may only be shared under the terms of a Data Use Agreement. Requests to access the datasets should be directed to weihong.zhuang@reckitt.com. The authors and study sponsor encourage and support the responsible and ethical sharing of data from clinical trials.
